# Surgical Management of Microstomia: A Comprehensive Review of Treatment Options

**DOI:** 10.1016/j.jpra.2025.03.002

**Published:** 2025-03-07

**Authors:** Daehee Jeong, Tara Sara Saffari, Amy Liao, Sara Iskeirjeh, Gary B. Skolnick, Justin M. Sacks, Saif Badran

**Affiliations:** a Division of Plastic and Reconstructive Surgery, Washington University School of Medicine, Saint Louis, Missouri; b College of Medicine and Public Health, Flinders University, Adelaide, Australia

**Keywords:** Microstomia, Surgery, Graft, Local flap, Free flap, Commisuroplasty

## Abstract

**Background:**

Microstomia is characterized by a reduced oral aperture, leading to impairments in speech, feeding, dental hygiene, and facial expressions. Nonsurgical treatments offer limited benefits for mild cases although surgical interventions have been shown to yield better outcomes. However, the unique anatomical challenges and limited availability of adjacent tissue present obstacles in surgical management of this condition. This review aims to provide a comprehensive summary of the reported surgical techniques for microstomia management and discuss their specific indications, advantages, and potential limitations.

**Methods:**

A comprehensive review was conducted in June 2024 to identify studies that have reported on the surgical techniques for microstomia management. A database search was performed on Medline, Embase, Scopus, and Web of Science. The study findings have been reported as per the Preferred Reporting Items for Systematic Reviews and Meta-Analysis guidelines. Quality assessment was conducted using the Case Report (CARE) guidelines and Methodological Index for Non‐randomized Studies (MINORS) assessment.

**Results:**

The search yielded a total of 1315 studies; of these, 28 met the inclusion criteria following a full-text screening. Ten surgical techniques for microstomia management were identified and categorized into the following three reconstructive modalities: grafts, local tissue transfer, and autologous free tissue transfer. The average scores of the CARE and MINORS assessments were 27.20/30 and 11.33/14 respectively.

**Conclusion:**

Selection of the appropriate surgical technique is critical for achieving successful results in microstomia reconstruction. The primary objective is to enlarge the oral opening while preserving oral function and achieving satisfactory cosmetic outcomes. The key considerations for choosing a surgical technique include contracture severity, perioral tissue availability, and the surgeon's familiarity with different surgical options.

## Introduction

The oral aperture plays a vital role in essential functions, such as speech, feeding, and cosmesis.^1^ Microstomia is a congenital or acquired deficiency in the oral aperture that can significantly impact oral form and function. Although microstomia is considered a rare disorder, it remains underreported. Studies indicate that 3.7%-10.8% of thermal burn cases and over 30% of diffuse facial scleroderma cases are complicated by microstomia.^2^^,^^3^ Pediatric populations are particularly vulnerable to this condition after experiencing physical trauma because their small oral aperture and predisposition to hypertrophic scarring contribute to contractile disfigurement of the mouth.^4^ Microstomia development is commonly attributed to scar contracture resulting from burns (flame or electrical), exposure to caustic agents, or surgical procedures.^5^^,^^6^ Rare causes include congenital malformations, such as Freeman-Sheldon syndrome, and connective tissue diseases, such as systemic sclerosis.^7^^,^^8^

Various surgical and nonsurgical treatment methods have been reported for microstomia management. Nonsurgical approaches are preferred for mild cases; these include splinting, pulsed light therapy, hyaluronidase, and botulinum toxin injections.^9^, ^10^, ^11^ These techniques offer advantages, such as simplicity and lower morbidity as compared to surgical approach; however, they have limited efficacy in patients with moderate to severe microstomia.^8^ Surgical reconstruction of the mouth involves unique challenges owing to the vermillion border, complex sphincter-like nature of the orbicularis oris muscle, and absence of a supportive skeletal structure.^12^^,^^13^ In order to overcome these challenges, various reconstructive options have been proposed. The selection of the appropriate procedure depends on several factors, including patient demographics, microstomia severity, and availability of adjacent tissue reservoirs. This comprehensive review aims to provide an overview of published surgical treatment options for microstomia management; provide the clinical context of these treatment options; and facilitate decision-making on the use of grafts, local tissue transfer, or autologous free tissue transfers.

## Methods

### Study inclusion and exclusion criteria

Studies that reported surgical treatment for microstomia were included. Studies involving nonsurgical treatments or surgical techniques aimed at addressing oral aperture issues unrelated to microstomia were excluded. In addition, abstract-only papers, non-English texts, and articles that focused on animal research were excluded.

### Search strategy

The search strategy followed the Preferred Reporting Items for Systematic Reviews and Meta-Analyses guidelines.^14^ A comprehensive literature search was conducted in June 2024, utilizing the following databases: Medline, Embase, Scopus, and Web of Science. Medical Subject Headings were employed, combining the terms “Microstomia” and “(Surgical Procedures, Operative OR Plastic Surgery Procedures OR surgery OR surgeries OR surgical OR operation OR operative OR postoperative OR reconstruction).” Two independent authors (D.J. and T.S.S.) assessed the eligibility of studies according to the predetermined inclusion/exclusion criteria. In cases where a consensus was required, discussions were held with the senior author (S.B.) to reach a mutual decision.

### Data synthesis

Data collected from each study included the first author's name, publication year, title, study design, reported outcomes, follow-up duration, and complications and have been presented in Table 1.^5^^,^^12^^,^^15^, ^16^, ^17^, ^18^, ^19^, ^20^, ^21^, ^22^, ^23^, ^24^, ^25^, ^26^, ^27^, ^28^, ^29^, ^30^, ^31^, ^32^, ^33^, ^34^, ^35^, ^36^, ^37^, ^38^, ^39^ The specific type of reconstruction employed, key indications for the reported technique, as well as the advantages and disadvantages of the techniques are included in Table 2.

**Table 1:** Overview of included studies.

Author (year)	Title	Study design (number of cases)	Reported technique	Outcomes	Follow up duration	Reported complications (n)	Quality Assessment Score
Hashem et al.^15^	Oral burn contractures in children	Case Series (3)	Skin Graft	MMO: 25-29 mm ICD: 20-27 mm	10-12 months		12/14
Zweifel et al.^12^	Management of microstomia in adult burn patients revisited	Case Series (4)	Skin Graft	-	30-33 months	Reoperation (2)	14/14
Gupta et al.^16^	Unusual presentation of caustic ingestion and its surgical treatment: a case report	Case Report (1)	Skin Graft	-	1 month		29/30
Ki et al.^17^	Reconstruction of microstomia considering their functional status	Case Series (1)	Skin Graft	MMO: 26 mm ICD: 36 mm	8 months		14/14
Ayhan et al.^18^	An alternative treatment for postburn microstomia treatment: Composite auricular lobule graft for oral commissure reconstruction	Case Series (2)	Composite Graft	MMO: 15-17 mm	24 months		12/14
Sawada et al.^19^	Expanding oral angle plasty using a subcutaneous pedicle flap to correct severe microstomia after extensive facial burns	Case Series (2)	Y-V Mucosal Advancement Flap	-	6 months		10/14
Mehra et al.^5^	Bilateral oral commissurotomy using buccal mucosa flaps for management of microstomia: Report of a case	Case Report (1)	Y-V Mucosal Advancement Flap	MMO: 30 mm	12 months		27/30
Mordijikian et al.^20^	Severe microstomia due to burn by caustic soda	Case Report (1)	Y-V Mucosal Advancement Flap	MMO: 35 mm ICD: 45 mm	6 months		25/30
Zweifel et al.^12^	Management of microstomia in adult burn patients revisited	Case Series (10)	Y-V Mucosal Advancement Flap	-	12-72 months	Reoperation (1)	14/14
Moorthy et al.^21^	Early Y-V commisuroplasty under local analgesia for burns induced microstomia	Case Report (1)	Y-V Mucosal Advancement Flap	-	-		22/30
Huang et al.^22^	Mucosal Y-to-V plasty for burn microstomia reconstruction in burn children revisited	Case Series (491)	Y-V Mucosal Advancement Flap	MMOAi: 108-1388 mm^2^	6-120 months	Reoperation (50)	12/14
Johns et al.^23^	The use of a triangular pedicle flap for oral commisuroplasty: Report of a case	Case Report (1)	Rhomboid Flap	ICD: 50 mm	6 months		27/30
Tuncer et al.^28^	Surgical treatment of a severe microstomia developed following leucocytoclastic vasculitis	Case Report (1)	Rhomboid Flap	-	84 months		27/30
Zweifel et al.^12^	Management of microstomia in adult burn patients revisited	Case Series (4)	Rhomboid Flap	-	20-26 months		14/14
Jaminet et al.^24^	Extreme microstomia in an 8-month-old infant: bilateral commissuroplasty using rhomboid buccal mucosa flaps	Case Report (1)	Rhomboid Flap	-	0.5 month		27/30
Turan et al.^25^	The use of single rhomboid flap in reconstruction of microstomia	Case Report (1)	Rhomboid Flap	ICD: 55 mm	8 months		27/30
Branch et al.^26^	Management of Severe Microstomia in a Ten-Week-Old Infant	Case Report (1)	Rhomboid Flap	-	1 month		28/30
Ki et al.^29^	Early surgical correction of microstomia following Stevens-Johnson syndrome	Case Report (1)	Rhomboid Flap (Lozenge)	MMO: 35 mm ICD: 41 mm	5-14 months		14/14
Kao et al.^30^	Posterior Based Triangular Mucosal Advancement Flap for Surgical Correction of Scleroderma-Induced Microstomia	Case Report (1)	Rhomboid Flap	MMO: 27 mm ICD: 49 mm	1 month		27/30
Monteiro et al.^31^	A simple “fishtail flap” for surgical correction of microstomia	Case Series (2)	Trapeze Flap (Fishtail)	ICD: 40-46 mm	6 months		10/14
Grishkevich et al.^32^	Post-burn microstomia: Anatomy and elimination with trapeze-flap plasty	Case Series (289)	Trapeze Flap	-	-		10/14
Mühlbauer et al.^33^	Elongation of mouth in post-burn microstomia by a double Z-plasty	Case Series (4)	Z-Plasty	MMOi: 20-25 mm			10/14
Sato et al.^27^	Surgical correction of microstomia in a patient with antilaminin 332 mucous membrane pemphigoid	Case Report (1)	Z-Plasty	IIDi: 50 mm ICD: 45 mm	24 months		8/14
Osaki et al.^34^	Commissuroplasty Using Split Dry Lips: A Case Report	Case Report (1)	Z-Plasty	-	6 months		29/30
Sari et al.^35^	Reconstruction of the oral commissure with the use of a new technique: The asterisk design	Case Series (6)	Asterisk	-	16.5 ± 16.3 months (Mean ± SD)		11/14
Makiguchi et al.^36^	Treatment of microstomia caused by burn with a nasolabial flap–an ingenious approach for tugging and fixation of the oral commissure	Case Report (1)	Nasolabial Pedicle Flap	MMO: 50 mm MMOi: 18 mm	12 months		28/30
Tavakoli et al.^37^	Paediatric oral caustic soda ingestion: Case series and lessons learned	Case Series (3)	Radial Forearm Free Flap	-	-		9/14
Manas et al.^38^	Management of Post-Electric Burn Microstomia by Free Radial Artery Forearm Flap in a 1-Year-Old Child	Case Report (1)	Radial Forearm Free Flap	-	6 months		29/30
Jin et al.^39^	Reconstruction of cicatricial microstomia and lower facial deformity by windowed, bipedicled deep inferior epigastric perforator flap	Case Report (1)	Deep Inferior Epigastric Perforator Flap	ICD – 60 mm ICDi – 34 mm	4 months		28/30

MMO: Maximal Mouth Opening. ICD: Intercommissural Distance. MMOi: Maximal Mouth Opening Improvement. IIDi: Interincisional Distance Improvement. MMOAi: Maximal Mouth Opening Area Improvement. SD: Standard Deviation. Quality assessment was conducted using the Case Report (CARE) checklist for Case Reports and the Methodological Index for Non‐randomized Studies (MINORS) assessment for Case Series.

**Table 2:** Overview of surgical techniques for the reconstruction of microstomia

Type of Reconstruction	Key Indications	Advantages	Disadvantages
Grafts			
• Skin Graft	• Mild microstomia • Minimal scar release required	• Effective for simple scar release • Effective for mucosal and cutaneous lesions	• Contracture over time • Potential aesthetic compromise
• Composite Graft	• Isolated commissural scarring	• Simple	• Lack mucosal replacement • Cannot significantly widen commissures • Poor tissue match • Risk of failure due to inadequate vascularization
Local Mucosal Flaps			
• Y-V Advancement Flap	• Localized pericommissural scarring	• Simple	• Requires transection of orbicularis oris muscle • Unsuitable if cutaneous scarring is severe • Risk of tension at the tip • Risk of tip necrosis • Risk of reformation of microstomia
• Rhomboid Flap	• Localized scarring	• Simple • Reduces tip necrosis risk	• Requires transection of orbicularis oris muscle
• Trapeze Flap	• Localized, superficial commisural scarring	• Preserves the orbicularis oris muscle	• Limited to superficial scarring cases • Minimal widening of intercommisural distance
Local Skin Flaps			
• Double Z-Plasty	• Localized scarring	• Simple • Preserves orbicularis oris muscle • Can be applied in multiple directions	• Limited ability to resect diffuse scar tissue • Limited ability to increase the oral aperture
• Asterisk Design	• Significant Diffuse scarring	• Significant scar tissue resection • Multiple flap vectors can increase the oral aperture in any direction • Reduces re-contracture risk	• Orbicularis oris must be incised if significant widening of commissures is needed
• Nasolabial Pedicle Flap	• Diffuse scarring • Microstomia with severe scarring	• Significant scar tissue excision • Preserves orbicularis oris muscle Minimizes ischemic failure risk	• May not significantly widen commissures • May place excessive tension on orbicularis oris muscle
Autologous Free Tissue Transfer			
• Radial Free Forearm Flap (RFFF)	• Very diffuse, deep, unilateral scarring	• Accommodates significant areas of scar tissue • Minimizes ischemic failure risk • Viable last-resort option	• May appear bulky • May require staged surgery • Reserved for complex cases
• Deep Inferior Epigastric Perforator (DIEP) Flap	• Very diffuse, deep, bilateral scarring	• Accommodates significant areas of scar • Suitable for bilateral defects • Minimizes ischemic failure risk • Viable last-resort option	• May appear bulky • May require staged surgery • Reserved for complex cases

### Quality assessment

A quality assessment was conducted by the first author (D.J.), according to the Case Report (CARE) guidelines and the Methodological Index for Non-Randomized Studies (MINORS) assessment for case reports and case series, respectively.^40^^,^^41^

## Results

**Figure 1:**
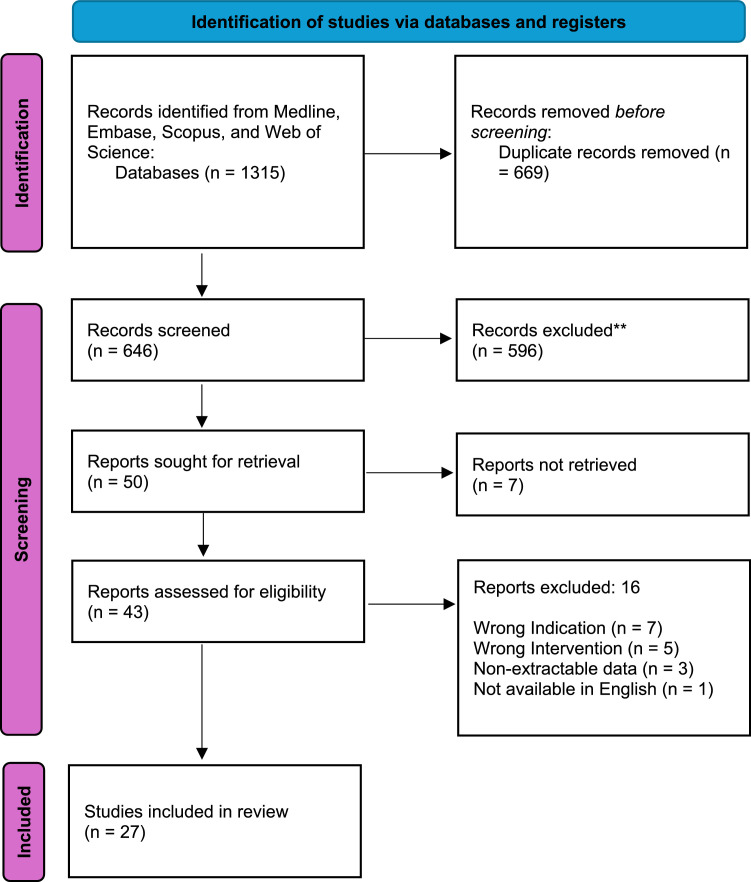
PRISMA flow diagram of article screening process. PRISMA: Preferred Reporting Items for Systematic Reviews and Meta-Analysis

The initial search using specific terms produced 1315 articles; of these, 669 duplicate articles were removed. After careful evaluation of the titles and abstracts, 597 studies were identified as irrelevant. The remaining 27 articles were found eligible for inclusion following a thorough assessment of the full texts (Figure 1).

The included studies discussed 10 surgical procedures that were further categorized into the following three groups: grafts, local tissue transfer, and autologous free tissue transfer. The following sections provide a comprehensive overview of established surgical techniques for microstomia management. This includes a discussion of the advantages, disadvantages, and indications for each procedure. Additional information is presented in Table 2.

The average score of the CARE checklist quality assessment for case reports was 27.20/30 (standard deviation = 1.78). The average score of the MINORS quality assessment for the case series was 11.33/14 (standard deviation = 2.02), as shown in Table 1. No statistical analyses of the outcomes were conducted between studies because the studies showed wide variation in reporting the type of quantitative outcomes, and some studies reported no quantitative outcomes. None of the studies presented standardized quantitative assessments of outcomes.

### Grafts

#### Skin grafts

Skin grafts are effective in addressing perioral scarring. The procedure relieves perioral tension by removing scar tissue or making incisions perpendicular to the lines of greatest tension. Skin is then harvested from a body area that is nonfibrotic, cosmetically neutral, and matching in color. The harvested skin is sutured over the resulting defect.^17^

Skin grafts may be suitable for treating mild cases of microstomia. However, it is important to note that these grafts tend to contract over time, potentially resulting in microstomia recurrence.^42^ In addition, aesthetic outcomes may be compromised owing to inherent differences between the graft and donor sites.^12^

#### Composite grafts

In 2006, Ayhan et al. reported the use of triangular wedge-shaped grafts from the ear lobule for commissure reconstruction in two cases of severe, diffuse perioral burns. The composite grafts were obtained by transecting the ear lobe, ensuring that the epidermis overlay the cartilage on both the sides. An incision was made from the edge of the lobule toward the graft tip, leaving the apex intact by 1 mm. The fibrotic commissure was excised and replaced with the composite graft.

This approach improved oral function and provided satisfactory cosmetic outcomes.^18^ However, composite grafts are criticized for their lack of mucosa, poor tissue match, and potential for hairiness in male patients.^25^ In addition, composite cartilage grafts involve a higher risk of failure due to inadequate vascularization through angiogenic vessels, resulting in significant donor site morbidity.^43^^,^^44^

### Local tissue transfer

The following reconstructive options utilize reservoirs of mucosal or local perioral cutaneous tissue, or both, for commissural scarring reconstruction.

#### Mucosal flaps

Mucosal flaps utilize oral mucosal surfaces for the reconstruction of the commissures after scar excision. These flaps have become popular owing to their simplicity and surgical efficacy.^12^

##### Y-V advancement flap

**Figure 2:**
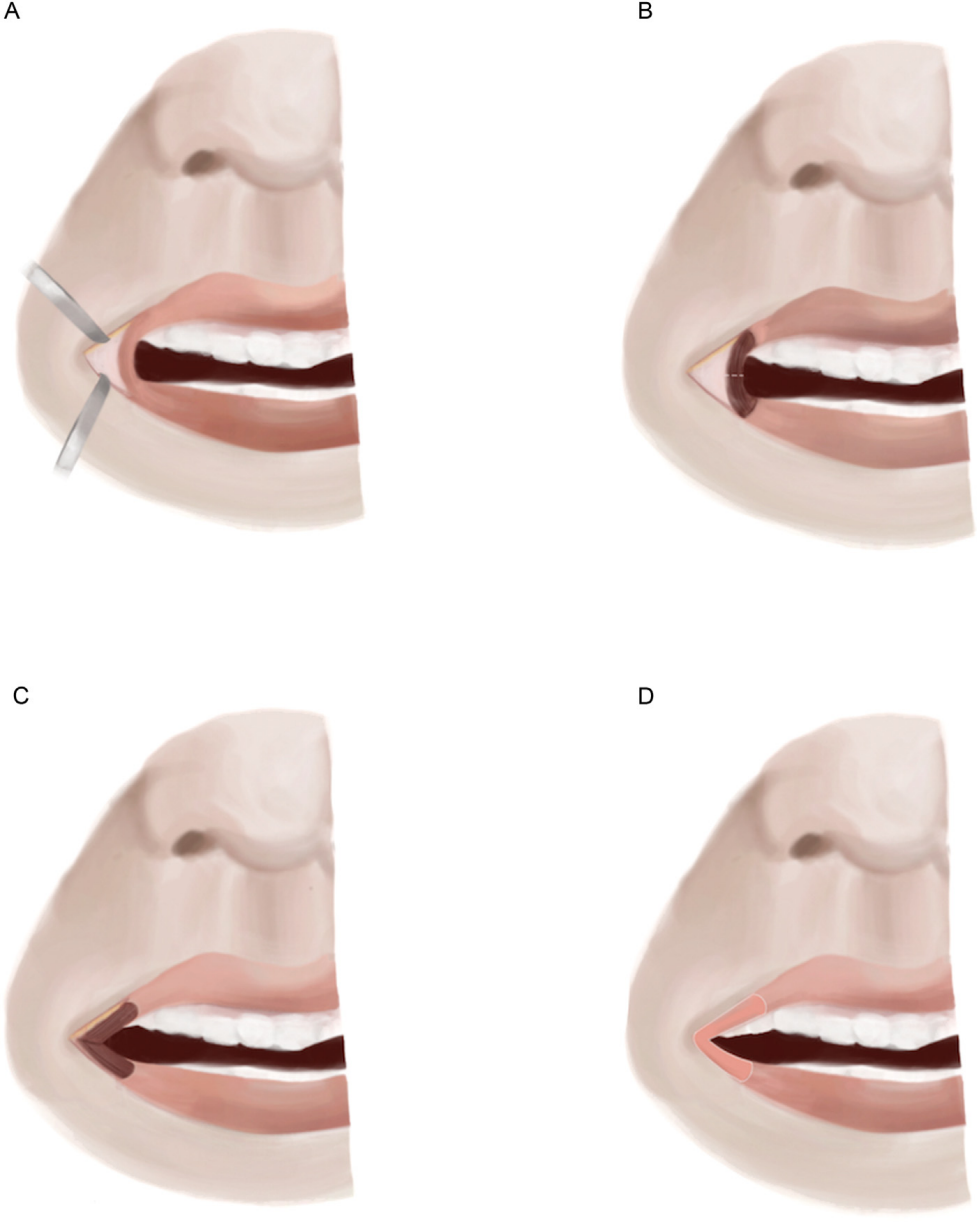
Y-V mucosal advancement flap. A) A full-thickness wedge-shaped incision extending to the lateral extent of the native commissure is performed. B) The vermillion border scar is excised, exposing the vertex of the orbicularis oris muscle. C) Following the division of the orbicularis oris muscle, the superior and inferior muscular flaps are lateralized and secured to the underlying subcutaneous tissue. D) The superior, lateral, and inferior borders of the buccal mucosa are advanced to restore the mucosa of the lips.

Y-V mucosal advancement flaps, initially described by Dieffenbach in 1831 and subsequently modified by Converse, involve excision of the scarred commissures using transverse wedge-shaped incisions extending to the desired new commissure locations. Thereafter, superior, inferior, and lateral triangular mucosal surfaces are advanced into place for each commissure (Figure 2).^45^

Y-V commissuroplasties are preferred for commissure reconstruction owing to their simplicity and favorable outcomes; however, the frequency of use varies among institutions and surgeons.^46^ Zwiefel et al. reported that 56% of patients requiring commissuroplasty between 1995 and 2007 underwent Y-V mucosal advancement flap procedures, while Grishkevich et al. reported a Y-V commissuroplasty use rate of only 16.2% from 1977 to 2010.^12^^,^^32^ These differences in usage frequency reflect the limitations of the technique, including tension at the mucosal flap tip and resultant necrosis, microstomia reformation, and inadequate removal of contractile scar tissue.^2^^,^^25^^,^^32^^,^^46^

##### Rhomboid flap

**Figure 3:**
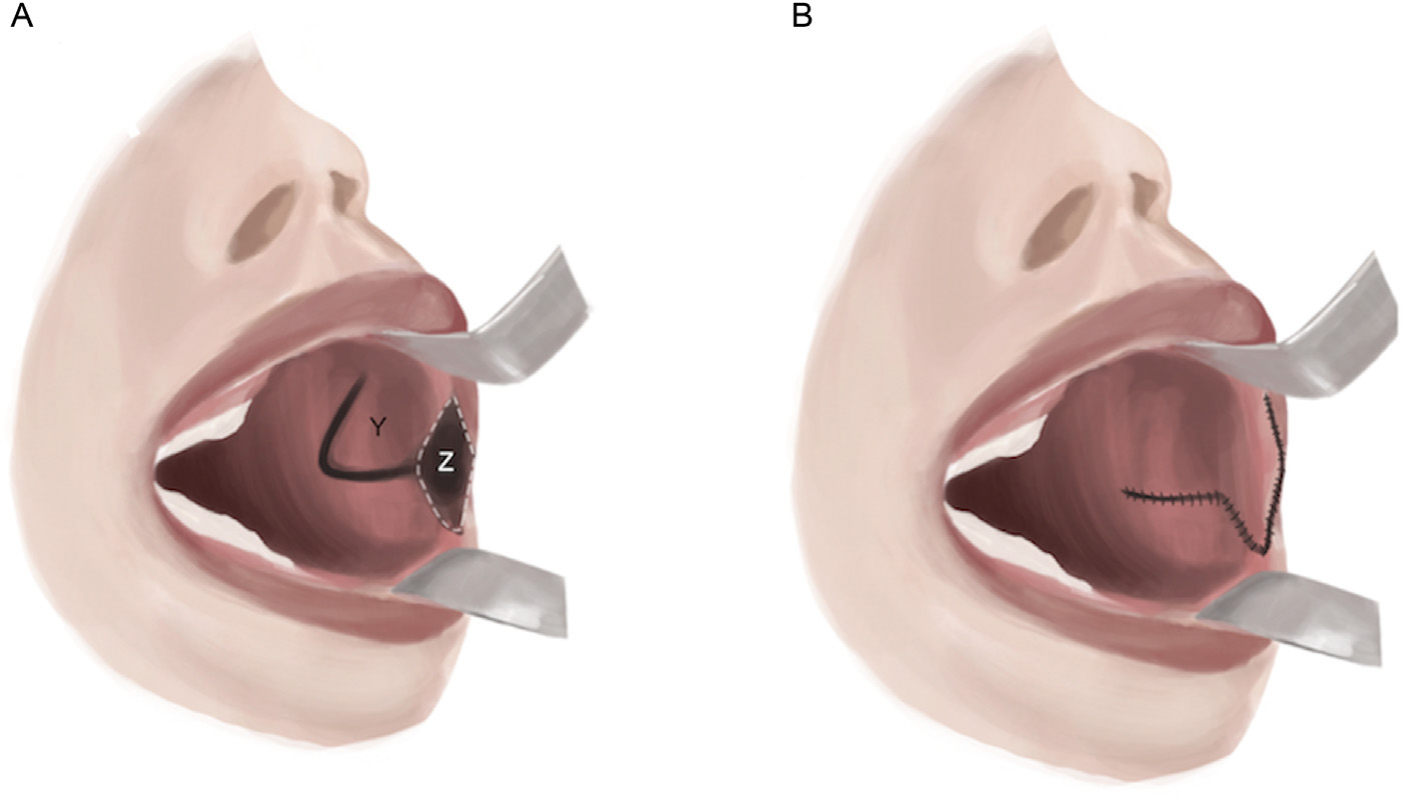
Rhomboid flap. A) A rhomboidal excision of the commissure resulting in wound bed Z is performed. B) The flap (Y) is transposed into the wound bed (Z) such that the distal end of the rhomboid becomes the neocommissural tip, and the entire distal edge of the flap forms the neocommissural vermillion border.

Rhomboid transposition flaps offer a distinct type of mucosal flap used for commissure reconstruction. First, the commissure is extended to the mid-pupillary axis with transverse skin incisions, and scar tissue is excised from the oral angles. Then, a rhomboid flap is elevated from the superior buccal mucosal surface and transposed such that the distal flap point becomes the neocommissural tip, and the entire distal edge of the flap forms the neocommissural vermillion border (Figure 3). In cases of severe microstomia due to oral angle scarring, a double rhomboid flap may be utilized from both, the superior and inferior tissue reservoirs. This modification has been reported in the treatment of patients with vertical oral apertures of 2 mm and horizontal oral apertures of 1 cm.^24^^,^^26^

Compared to Y-V plasty, the rhomboid flap provides a major advantage of placing maximum tension on the flap base rather than on the tip, reducing the risk of tip necrosis.^47^ However, the rhomboid flap has a significant limitation: the need to transect the orbicularis muscle for commissural extension.

##### Trapeze flap

**Figure 4:**
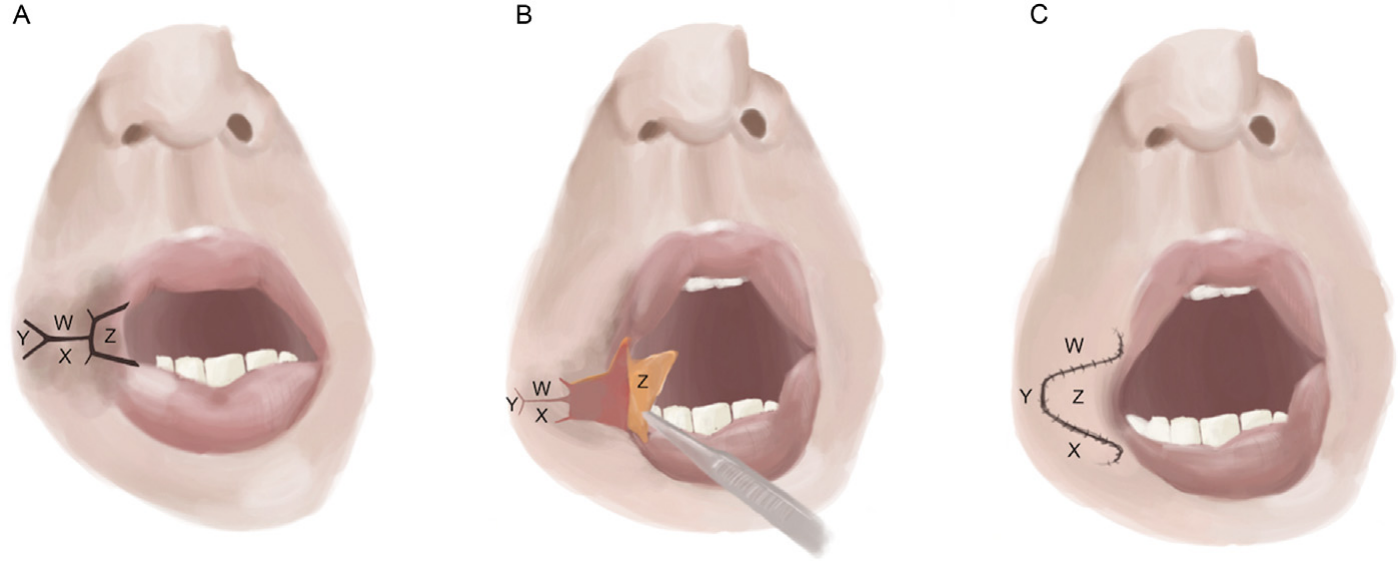
Trapeze flap. A) An initial transverse Y-shaped cutaneous incision is made lateral to the oral commissure, forming a trapeze-shaped wound. B) Superficial scar tissue is excised from the orbicularis oris muscle to widen the oral aperture. A trapezoidal flap is created on the oral mucosal surface, lateral to the initial incision and is prepared for advancement. C). Lateral advancement of flap Z, positioned inferiorly to flap W and superiorly to flap X.

The trapeze flap involves an initial transverse Y-shaped cutaneous incision, similar to the Y-V flap, forming a trapeze-shaped wound on the cutaneous surface lateral to the commissure. Superficial scar tissue is excised from the orbicularis oris to further widen the oral aperture. A trapezoidal flap is created on the oral mucosal surface lateral to the initial incision, advanced over the wound, and sutured into place (Figure 4). Grishkevich et al. have described a case series of 345 patients who were treated from 1977 to 2010; 289 patients underwent trapeze flap commissuroplasty for microstomia.^32^ Another similar flap, known as the “fishtail” flap, was introduced in 2011 by Monteiro et al.^31^

The trapeze flap offers the advantage of preserving the orbicularis muscle by advancing the mucosa laterally over the muscle to extend the commissure instead of cutting across the muscle. The trapeze flap is best suited for patients with superficial scarring rather than those with local musculature involvement in the scar tissue.^32^ It is noteworthy that using only transverse incisions at the commissure primarily relieves skin tension in the superior-inferior direction without increasing the intercommissural distance.

#### Skin flaps

Several different cutaneous or myocutaneous flaps have been reported that utilize both, the perioral cutaneous tissue and oral mucosa for reconstruction.

##### Double Z-plasty

In 1970, Muhlbauer introduced the double-opposing Z-plasty technique for microstomia, which involves two symmetrical Z-plasties with central axes placed along the vermillion border and across the horizontal axis of the commissure.^33^ A Z-plasty is a surgical technique that creates a Z-shaped incision to redistribute tension and lengthen tissue. Each Z-plasty limb involves the vermillion and mucosa as well as the adjacent cutaneous tissue. Transposing these limbs lengthens the wound, increasing the oral opening. The double Z-plasty offers several advantages, such as avoiding transecting the orbicularis oris, the ability to perform the procedure under local anesthesia, and its applicability in multiple directions.^33^ However, it is rarely utilized owing to its insufficient ability to restore the oral angle in patients with diffuse scarring.^48^

##### Asterisk design

The Asterisk design, described by Sari et al. in 2009, involves a superficial transverse incision from the commissure to the desired location of the neocommissure with preservation of the underlying orbicularis oris muscle. Two triangular skin flaps (A and B), one superior and one inferior, bordering the vermillion and with their apex at the original commissural site, are elevated. Thereafter, triangular standing tissue cones located laterally to flaps A and B are excised. Then, the orbicularis oris muscle is incised while preserving the underlying mucosa, which is advanced laterally to the new commissural location, crossing over the intact orbicularis to form a posterior-based mucosal flap. The two vermillion borders, along with flaps A and B, are then advanced laterally to meet at the neocommissure, replacing the removed standing tissue cones.^35^

The advantage of the Asterisk design is its utilization of multiple smaller flaps oriented in different directions, distributing skin tension in multiple vectors and reducing the recontracture risk. This design allows for an increase in the oral opening in any direction. However, similar to other described flaps, it extends the commissure laterally by incising the orbicularis oris muscle, compromising the integrity of the oral sphincter.

##### Nasolabial pedicle flap

**Figure 5:**
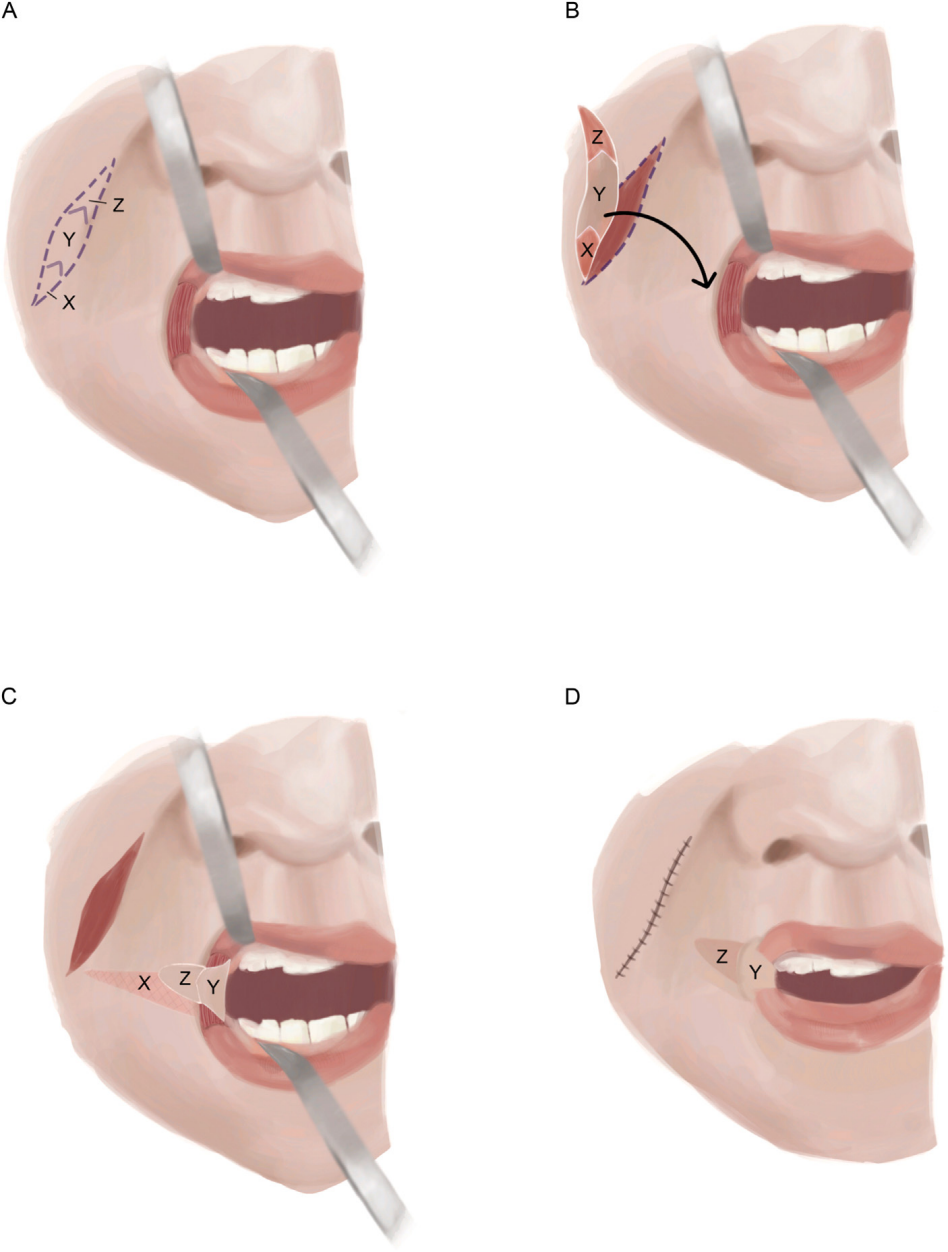
Nasolabial pedicle flap. A) A full-thickness excision of the scarred commissure is performed, preserving the underlying orbicularis oris muscle. The nasolabial pedicle flap is mapped in a trapezoidal shape. B) The pedicle is elevated, and epithelial denudation of segments X and Z is performed. C) The pedicle is tunneled through the subcutaneous pocket, which extends from the inferior base of the nasolabial fold to the orbicularis oris muscle, looped around the orbicularis oris muscle from the inferior plane, and positioned laterally to cover the trapezoidal defect. D) The pedicle site is adjusted such that the epithelium of segment Y overlays the defect and is secured superficially. The distal denuded portion (segment X) is anchored subdermally, followed by a linear closure of the nasolabial incision site.

In 2014, Makiguchi et al. described a surgical technique for microstomia that uses a tunneled pedicle flap from the nasolabial fold. The scarred commissure is excised in a trapezoidal shape with preservation of the underlying musculature, with the wider base positioned at the medial border of the oral angle. The entire orbicularis oris muscle is elevated from the underlying fascia and remains preserved. A subcutaneous pocket is extended superolaterally from the orbicularis to the inferior base of the nasolabial fold. A pedicle in the shape of a bipointed ellipsoid, with the base at the inferior nasolabial fold, is raised along the nasolabial fold. The pedicle is denuded of the epithelium, except for the middle third, that will cover the trapezoidal defect. Then, the pedicle is tunneled through the subcutaneous pocket, looped around the orbicularis oris from the inferior plane, and positioned laterally to cover the trapezoidal defect, with the remaining epithelium of the pedicle overlaying the defect. The pedicle can be adjusted by pulling it laterally against the orbicularis oris to ensure the precise placement of the neocommissure. The denuded portion of the pedicle at the distal end is anchored subdermally, while the rest of the pedicle is secured superficially. Finally, the nasolabial fold may be closed linearly (Figure 5).^36^

The nasolabial pedicle flap is advantageous in that it allows for significant scar excision, increasing the size of the oral opening and improving functional outcomes. In addition, preservation of the entire orbicularis oris muscle ensures that oral sphincter function is maintained if excessive tension is avoided. Finally, the robust vascularity provided by the facial artery minimizes the risk of ischemia.^49^ However, this technique may be unsuitable for microstomia patients who require significant intercommissural distance lengthening because it may create excessive tension on the orbicularis oris.

### Autologous free tissue transfer

In patients of severe microstomia, such as that caused by burns, diffuse scarring could make local flap reconstructions insufficient for alleviating skin tension, and local tissue reservoirs may be too fibrotic for successful reconstruction. The soft tissue reconstruction approach depends on the defect size and involved structures (skin, muscle, or mucosa). Several options have been reported for free tissue transfer in lip reconstruction, including fasciocutaneous flaps (such as anterolateral thigh flaps and superficial circumflex iliac artery perforator flaps) and muscle flaps, including innervated muscle flaps (such as the gracilis and latissimus dorsi muscles).^50^, ^51^, ^52^, ^53^ This section presents two examples of autologous free transfer flap utilization for reconstruction in severe and extensive cases of microstomia involving both the upper and lower lips. These examples illustrate the potential use of free flaps and how the incorporation of the aforementioned local flaps can complement free flap surgery.

The radial forearm free flap (RFFF), initially described by Yang et al. in 1981, is commonly employed for reconstructing defects in the oral cavity and the oro-hypopharynx.^54^^,^^55^ The RFFF procedure is performed in two stages. In the first stage, a suprafascial forearm flap is raised, including the radial artery pedicle and the cephalic vein. Anastomoses are established between the radial artery and the recipient facial artery, as well as between the cephalic vein and the external jugular vein. The second stage is performed approximately two months thereafter; horizontal incisions are made to release the new oral angle, and the mucosa is advanced to the flap borders to recreate the commissure, upper lip, and lower lip. In some cases, the flap may be debulked at this stage.^38^ The RFFF is suitable when local tissues cannot be utilized owing to extreme microstomia with diffuse scarring. It supplies its microvasculature through the anastomoses with the facial artery, reducing the risk of flap failure because of ischemia, especially in patients with compromised local vasculature secondary to extensive tissue damage. Therefore, the RFFF serves as a viable option when alternative reconstruction methods could be ineffective. An example of RFFF application has been reported in the case of a 1-year-old with severe unilateral microstomia following an electrical burn. It is noteworthy that in this case, only one RFFF was required, enabling the contralateral side of the mouth to remain open for feeding during the interstage period.

For more severe bilateral cases that require additional lower face reconstruction, additional surgery stages or alternative options may be considered, such as the deep inferior epigastric perforator (DIEP) flap. Jin et al., in 2009, described microstomia treatment in a patient with severe scarring on the lower half of the face following a flame burn, utilizing a DIEP flap with a full-thickness window. In order to perform this procedure, a wound bed is first created on the lower face, followed by identification and measurement of the oral fissure. The integrity of the orbicularis oris muscle is confirmed. Then, bilateral inferior epigastric vessels are anastomosed with facial vessels. A centrally positioned transverse incision, corresponding to the width of the oral fissure, is made over the desired location of the new oral aperture, creating a window within the tissue. The oral mucosa is then elevated and advanced circumferentially to the flap borders for reconstruction of the lips and oral commissures.^39^ The windowed DIEP flap finds suitability in cases of bilateral scarred defects that lack other reconstructive options. The windowed DIEP shares certain benefits and limitations with the RFFF; however, the windowed DIEP flap is more suited for patients with bilateral defects, while the RFFF is more appropriate for unilateral defects.

## Discussion

Due to the intricate structure of the oral aperture, managing microstomia poses a substantial reconstructive challenge. The optimal approach depends on several factors, including the severity of microstomia, the degree and focal location of perioral scarring, and the quality of the local vasculature. Surgical options for microstomia involve grafting, local tissue transfer, and autologous tissue transfer.

Skin grafts are typically employed for less complicated cases where scarring is limited to the superficial layers and does not extend past the underlying fascia, allowing correction via superficial contractile scar release. These reconstructions can be conducted rapidly; however, they may contract over time. In addition, when scarring affects multiple anatomical layers of the oral aperture (e.g., mucosa, lips, commissure), a single skin graft may not address the distinct qualities of each region, potentially compromising functional and cosmetic outcomes.

Local tissue transfer may be considered for microstomia cases involving more diffuse fibrosis. One advantage of this approach is the wide variety of available techniques, matching the highly variable presentation of microstomia. For example, mucosa-based flaps, such as the Y-V advancement and rhomboid flaps, are well-suited for releasing contracture caused by intraoral etiologies, such as caustic ingestion. In contrast, a cutaneous local flap may be better suited for microstomia resulting from external thermal burns or cutaneous cancer resection. In addition, specialized local flap techniques can treat microstomia involving specific oral structures, such as the orbicularis oris, vermillion borders of the lips, or commissures. For example, Z-plasties, as described by Muhlbauer, are particularly effective for releasing scarring along the oral commissure while concealing incisions along the vermillion border.^33^

Autologous free tissue transfer is sometimes indicated for microstomia cases that involve extensive damage to the perioral tissue that compromises local vascular viability. In such cases, the flaps depend on an uncompromised donor vascular network to ensure flap survival. However, these techniques should be used sparingly as needed and may result in suboptimal cosmetic outcomes despite debulking reoperations.^38^^,^^39^ Alternative approaches may be explored in these extreme instances. The initial step involves excising the fibrotic and scarred tissue, followed by applying the principles of lip reconstruction based on the size and anatomical layers of the defect. Although there is extensive literature on the use of fasciocutaneous and muscle free flaps, including innervated muscle flaps, in lip reconstruction, reports of free flap usage in microstomia cases are scarce. This could be attributed to the relative rarity of such cases compared to traumatic injuries and postmalignancy excision defects. However, the reconstruction principles for defects resulting from microstomia scarred tissue excision can be similar to those used in lip reconstruction, potentially combining several techniques, such as grafts, local tissue transfer, and free flaps simultaneously.

This review represents the first attempt to evaluate the available surgical options for microstomia management and provide a practical framework for surgeons to select an appropriate treatment option. The review aimed to encompass all published techniques potentially applicable to microstomia treatment; however, the authors acknowledge that a comprehensive list of techniques used for lip reconstruction would be highly beneficial in managing microstomia patients. For instance, techniques such as the facial artery musculomucosal flap and the submental flap have been described in oral reconstruction, although not specifically indicated for microstomia.^56^^,^^57^ Similarly, autologous tissue transfers such as the anterolateral thigh flap and gracilis flap, though not yet reported for microstomia treatment, remain viable options for oral reconstructive surgery.^50^^,^^52^ Nonetheless, a comprehensive understanding of currently reported surgical techniques and indications is crucial for enabling well-informed decisions in microstomia treatment and the refinement of new, optimized techniques. Another limitation includes the variability in reported outcomes among the included studies, which can be attributed to differences in patient populations, microstomia severity, and the level of surgical expertise. While some studies have reported on the final cosmetic and functional results, few have provided quantifiable measurements of microstomia improvement, and none have adopted standardized cosmetic outcome assessments, such as the Patient And Observer Scar Assessment Scale.^58^ Further, long-term outcomes and potential complications associated with each technique were not consistently reported across studies, limiting the ability to conclude the comparative efficacy of these techniques. Future studies should aim to incorporate consistent and standardized outcome measurements to assess the efficacy of surgical treatments for microstomia.

## Conclusion

Reconstruction of microstomia poses significant challenges to reconstructive surgeons, considering the complexity of the oral anatomy. Moreover, the variability in microstomia presentations necessitates an individualized approach to determine the most suitable surgical technique for each case. Key principles in selecting the appropriate reconstruction method include the extent of contracture, the availability of perioral tissue reservoirs, and the viability of reconstructive flaps. Careful selection of the appropriate reconstruction technique can enable surgeons to effectively expand the oral aperture with concurrent preservation of oral function and achievement of satisfactory aesthetic outcomes.

## Funding

None.

## Conflicts of interest

JMS is a co-founder of the Johns Hopkins Biotech startup Lifesprout. The remaining authors have no conflicts of interest to disclose. All the authors declare that the research was conducted in the absence of any commercial or financial relationships that could be construed as a potential conflict of interest.
